# Humanoids Learning to Walk: A Natural CPG-Actor-Critic Architecture

**DOI:** 10.3389/fnbot.2013.00005

**Published:** 2013-04-08

**Authors:** Cai Li, Robert Lowe, Tom Ziemke

**Affiliations:** ^1^Interaction Lab, University of SkövdeSkövde, Sweden

**Keywords:** reinforcement learning, humanoid walking, central pattern generators, actor-critic, dynamical systems theory, embodied cognition, value system

## Abstract

The identification of learning mechanisms for locomotion has been the subject of much research for some time but many challenges remain. Dynamic systems theory (DST) offers a novel approach to humanoid learning through environmental interaction. Reinforcement learning (RL) has offered a promising method to adaptively link the dynamic system to the environment it interacts with via a reward-based value system. In this paper, we propose a model that integrates the above perspectives and applies it to the case of a humanoid (NAO) robot learning to walk the ability of which emerges from its value-based interaction with the environment. In the model, a simplified central pattern generator (CPG) architecture inspired by neuroscientific research and DST is integrated with an actor-critic approach to RL (cpg-actor-critic). In the cpg-actor-critic architecture, least-square-temporal-difference based learning converges to the optimal solution quickly by using natural gradient learning and balancing exploration and exploitation. Futhermore, rather than using a traditional (designer-specified) reward it uses a dynamic value function as a stability indicator that adapts to the environment. The results obtained are analyzed using a novel DST-based embodied cognition approach. Learning to walk, from this perspective, is a process of integrating levels of sensorimotor activity and value.

## 1 Introduction

In recent years, with increasingly reforming ideas about how locomotion should be understood in a way that it is a result of the interaction of dynamical systems, bio-inspired approaches are attracting a lot of attention. Scientists claim that locomotion including its development or adaptivity emerges when the neural structure or the body with proper morphology interacts with the environment under the laws of physics (Pfeifer and Bongard, 2006; Ijspeert, 2008). Hence, the focus of investigating locomotive capabilities of artificial or biological agents should be shifted from how each body part moves in a kinematic chain to a generic view pertaining to how controllers (or neural systems), body, and environment interact as a *complete dynamic* *system*.

Recently, cutting-edge work in robotics shows the importance of the abovementioned ideas. According to Ijspeert, Central Pattern Generators (CPGs), the bio-inspired neural structures discovered in the middle of the last century (Hooper, 2001), work as a link connecting the sensori-motor level to the Mesencephalic Locomotor Region (MLR) in the brainstem which controls vertebrate locomotion. Thus, many robots under control of CPGs show their own adaptive behaviors when interacting with the environment (Fumiya et al., 2002; Pfeifer and Bongard, 2006; Degallier et al., 2011). A CPG network is a neural controller which can show adaptive network behaviors given sensory feedback. On the other hand, body flexibility, namely the so-called soft robotics, has been highlighted recently as a critical element for adaptive motor capabilities (Pfeifer and Bongard, 2006). However, there is no systematic way of evaluating flexibilities of different morphologies for locomotion.

On this basis, learning locomotion becomes more open and challenging in terms of integrating interactive information amongst the three parts: controllers, body, and context. Based on the *dynamic systems approach* proposed by Thelen in the 1990s from the perspective of development of cognition and action, locomotion is a consequence of self-organization and there is no “essence” for locomotive systems. Learning to walk is a formation process of a gait attractor dependent on the exploration of the state space in a dynamical system that consists of sensori-motor coupling of agent and environment. The attractor is a behavioral mode and *state space* is an abstract construct of space whose coordinates define the degrees of freedom of the system’s behavior (Thelen and Smith, 1996). However, the learning mechanism which causes the formation of an attractor out of the state space in artificial systems still remains unclear in spite of Thelen’s embodied theoretical stance. Adolph et al. (2012) posits that infants learn to walk through thousands of time-distributed, variable attempts including missteps and falls. She emphasizes the importance of the temporal-difference in the learning process. From the cognitive perspective, Schore (2012) indicates affective modulation is important for infants learning to walk. Particularly, the main caregiver plays a role as an “emotion system” outside assisting infants to evaluate their behaviors and scaffolding their affective systems. Pfeifer and Bongard (2006) explains locomotion learning from a robotics angle suggesting there is a “value” system in our body to evaluate the comfort of locomotion behaviors. Therefore, we assume there is an agent-centered mechanism related to learning how to walk and it has to comprise these properties: (1). It is an interactive-affective system. (2) It is capable of finding an optimized solution by exploring the state space through interaction with the environment in a time-sensitive manner. (3) The learning process is under control of the supervisor’s “scaffolding.” *We suggest, closely pertinent to* *the above three points, that reinforcement learning is an* *appropriate choice for the implementation of learning to* *walk*.

Reinforcement learning (RL) has, in recent years, evolved considerably especially in dealing with problems of continuous and high-dimensional state space (Doya, 2000b; Wiering and van Otterlo, 2012). Biologically, it sketches an interactive process of dopamine systems and the basal ganglia which is emotion-related (Schultz, 1998; Doya, 2000a; Graybiel Ann, 2005; Khamassi et al., 2005; Frank and Claus, 2006; Joel et al., 2012). Grillner et al. (2005) elucidate the functions of dopamine systems (striatum) and the basal ganglia (pallidum) with biological grounds on motor adaptation and selection. Moreover, RL proffers a computational formulation of learning, via the interaction of body, neural systems, and environment, to execute behaviors that deliver satisfying consequences. Grillner et al. (2007) also propose a layered architecture including basal ganglia, CPG network, and sensory feedback which may imply the interactive bond between CPGs and RL. In this article, by using RL, a meaning of “scaffolding” is given by manipulating the value function and update rules. Meanwhile, for the purpose of endowing a humanoid with a capability of learning to walk efficiently, the RL algorithm has to guarantee fast convergence.

Based on the above ideas and theories we propose a new architecture combining Natural Actor-Critic (NAC) and a CPG network to achieve a “learning to walk” task on a humanoid. This is the so-called Natural CPG-Actor-Critic. The natural actor-critic has been proposed by Kakade (2002) and further improved and used by Peters in the field of supervised motor learning (Peters and Schaal, 2006, 2008). This particular RL algorithm uses natural policy gradient methods which may achieve very efficient exploration and fast convergence of learning. Based on their ideas, Nakamura et al. (2007) proposed a natural CPG-Actor-Critic approach and implemented it with a 2*D*^1^-simulated stick walker in MATLAB. At the present time, the natural CPG-Actor-Critic has not been implemented on a humanoid platform. The reasons are clear: firstly, there exists no functional 3D CPG walking model that does not depend on inverse kinematics even though the motion of roll direction is of importance to walking (Collins et al., 2001). Nakamura’s work fully adopted Taga’s model (Taga, 1998) which similarly works on a 2D-simulated stick walker. Secondly, Taga’s model is very complicated involving a very high-dimensional and difficult-to-reduce state space. This is why state value estimates take a long time to converge. Finally, the stick walker contacts the ground in an entirely different way to humanoids with foot interaction so that the body dynamics also differ. This is a morphology-related reason. Thus, in this article, we try to use another sensor-driven CPG architecture to avoid the problems faced by Nakamura and colleagues (For the comparison to Nakamura’s model, please refer to Discussion A.1).

The main contribution of this article is to present a complete natural CPG-Actor-Critic architecture and implement it on a 3D-simulated humanoid by utilizing a state-of-the-art natural policy gradient in a relatively high-dimensional state space. In this work, it is shown not only how episodic NAC (eNAC) converges to optimal solutions by exploration-exploitation batch learning but also how eNAC helps a humanoid under control of CPGs learn to walk by searching appropriate posture and integrating sensory feedback. Meanwhile, by adopting a dynamic system perspective with respect to cognitive development, RL can be understood in a new light of state value estimates. Experiments introduced in this article consist of two parts. The first part will focus on the emergence of proper walking posture and integration of sensory feedback. The second part shows how the robot learns to walk on a slope and the relation between slope and posture change. The aim of this work is to glean how CPGs in a natural actor-critic architecture adapt to the environmental change in walking by balancing realization of body morphology and acquisition of sensory feedback.

## 2 Materials and Methods

In order to fully comprehend how CPG networks work with the NAC architecture, a description of relevant theories applicable to the proposed architecture is offered in this section. With the cpg-actor-critic model, it is able to clearly show how the humanoid’s body, the physical world, and neural controllers interactively cause the emergence of an appropriate walking gait. In order to learn walking, a proper upright standing posture is necessary. Scientific research shows that human infants learn to walk after they have learned to be able to maintain an upright posture (Kail and Cavanaugh, 1996; Adolph et al., 2012). After learning a standing posture, they can start to explore the world in an allocentric way. Through exploration, infants improve their walking behaviors (Clearfield, 2011). However, the exploration in a physical world consists of infinite possibilities increasing the difficulties in modeling this process. Thus, a limited but continuous state space has to be constructed for the purpose of learning to walk by exploring only in the state space of neural structure which is related to posture control and sensory feedback. Then walking can be considered as a Partially Observable Markov Decision Process (POMDP). In this article, we use a NAC architecture which appears as one good solution to bridge continuous state space and action space in a fast-learning way. We show that it can not only show the emergence of proper walking posture but also adaptation to environmental changes.

### 2.1 Central pattern generators

Modeling walking on a humanoid robot is a complicated task related to designing an autonomous control mechanism for a high degree-of-freedom (DOF) body. So the main challenge for developing modern control strategies concerns avoiding the problem of the “curse of dimensionality” which closely pertains to a large number of DOFs. Using CPGs, it is possible to transfer and restrict extremely high-DOF walking in Cartesian space to a low-dimensional sensory space of neural structure with neurophysiological theories and assumptions (Geng et al., 2006; Takamitsu et al., 2007; Endo et al., 2008).

CPGs, as a group of presumed neurons existing in vertebrates’ spinal cord (Latash, 2008), are the neural circuits generating rhythmic movement. With sensory feedback, the body or the robot under control of CPGs interacts with the environment in an adaptive way in which case the body dynamics are interactively entrained into a limit cycle. This limit cycle implies the following: firstly, structural-stability is imperative to a CPG architecture. This means CPG architectures should be able to shift to another limit cycle by adapting to contextual change and then recovering the original limit cycle without external disturbance (Righetti, 2008; Li et al., 2011). Secondly, the adaptive change of the limit cycle that CPGs converge to is generally done by updating the output or connection weights of CPGs. A lot of work has been done to emphasize the importance of these two points (Inada and Ishii, 2004; Ijspeert, 2008; Li et al., 2011, 2012).

Compared to a lot of work done with engineering models based on Zero Momentum Point (ZMP) (Lim et al., 2002; Strom et al., 2009) to model walking, CPGs also have many advantages (Nakamura et al., 2007). In terms of adaptive capabilities, as engineering models (including an accurate model of the controlled system and the environment) need to calculate the trajectories of motion with respect to very specific models, these models need to be recalculated or even remodeled when the context or the body changes. But, as for CPGs, it is just a matter of updating parameters to new adaptation capabilities. On the other hand, CPGs are proven to be more energy-efficient (Li et al., 2011) than those methods which need huge computer power to calculate complicated accurate models in each computation period.

From the perspective of the dynamic systems approach, just because of the excellent adaptivity of a CPG or its network, CPGs can be considered as an interface between the environment and high-level cognitive functionalities. As abovementioned, the shift and change of limit cycles could be viewed as results of CPGs interfacing to the high-level control system, like the RL system in this work.

#### 2.1.1 Layered CPG structure

**Figure 1:**
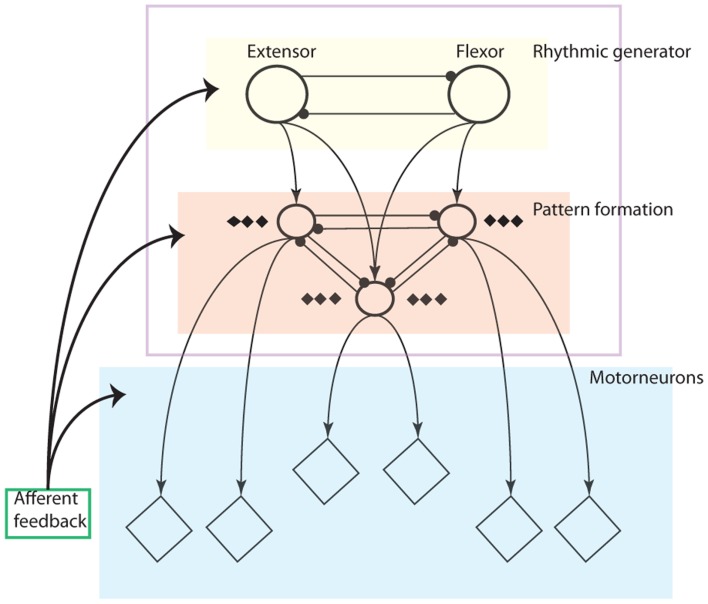
**Schematic illustration of the three-level central pattern generator (CPG) concept: The locomotor CPG consists of a half-center rhythm generator (RG), a pattern formation (PF) network and a motorneuron layer**. Rhythmic generator layer (yellow area): this layer contains oscillators which generate rhythmic signals as the input to the PF layer. PF layer (red area: only three neurons are drawn with others neglected): The PF network contains interneuron populations, each of which provides excitation to multiple synergistic motorneuron pools (diamonds) and is connected with other PF populations via a network of inhibitory connections. It mediates rhythmic input from the RG to motorneurons and distributes it among the motorneuron pools. The network also synchronizes the oscillatory output of each interneuron. The motorneuron layer: It integrates the muscle sensory feedback and activation of PF network outputs. The extensor and flexor motorneurons together determine the output to the muscles (Rybak et al., 2006).

CPG structures have been explored by researchers for some time (Orlovskii et al., 1999; Amrollah and Henaff, 2010) but the integration of sensory feedback remains an unresolved open question to the research of CPGs without a conclusive structure. Recently, a proper layered CPG architecture has been proposed in Rybak et al. (2006) based on biological evidence (Amrollah and Henaff, 2010; Figure 1).

The layered CPG concept illustrates clearly not only the functions for each layer but also principles for the influence of afferent feedback in each layer. For instance, the rhythm generator (RG) layer is in charge of rhythm or frequency resetting depending on feedback. The PF layer functions like a network to keep synchronization of motorneuron activities as well as phase transition without altering the RG layer according to afferent feedback. The motorneuron level is an integrator where downward outputs and sensory feedback are fused together (details in Figure 1).

**Figure 2:**
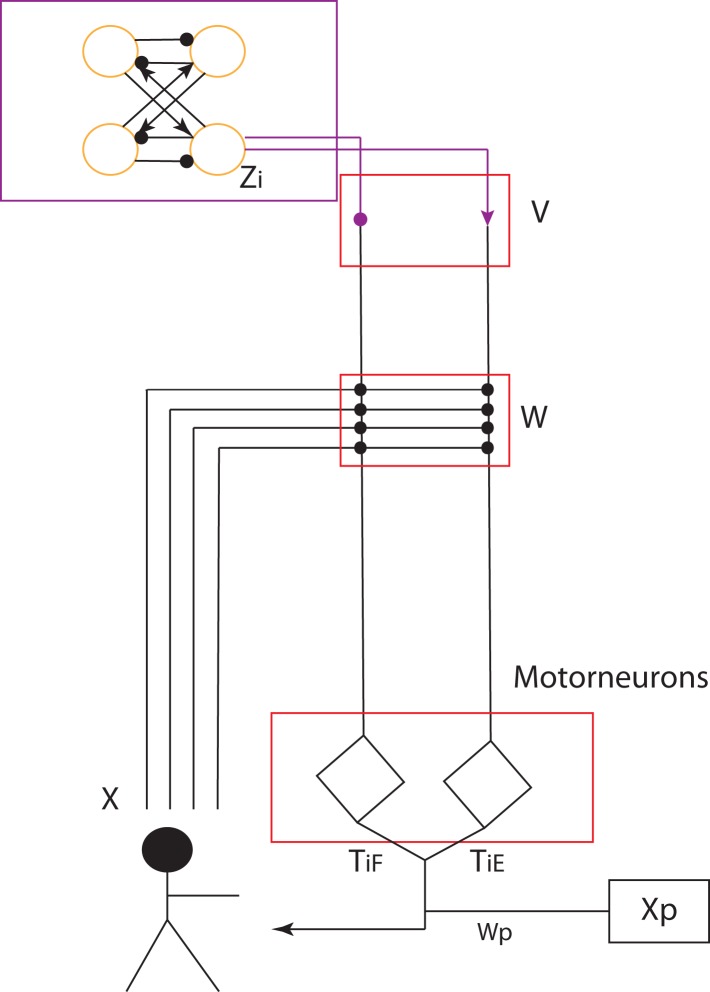
**CPG controller (Top: the four-cell network) and its layered structure**. Yellow circles represent a coupled RG group corresponding to yellow area in Figure 1. The round-headed and sharp-headed arrows represent negative (−1) and positive (+1) connection weights (for details, please refer to text.) The four-cell network (purple-framed area) fulfills the function of the PF layer. The two diamonds represent the motorneuron layer which integrates sensory feedback and upper-layer outputs. *V*, *W*, *W_p_* are weight vectors which integrate PF-layer outputs, sensory feedback and posture control terms respectively. *T_Ei_* and *T_Fi_* are the strength weights of extensor and flexor.

Based on this CPG structure, we propose a layered CPG architecture in our work which fulfills functions of each layer (Figure 2). In the structure, the four-cell recurrent network based on symmetric group theory (Golubitsky and Stewart, 2004) has the capability to be structurally stable (Righetti, 2008). It is of importance that this network can model the dynamics of different locomotion gaits (including walking, trotting, running, and crawling) by altering its connection weights and properties of each cell (Righetti, 2008). Crawling and walking on different humanoids have been implemented (Righetti and Ijspeert, 2006; Lee et al., 2011; Li et al., 2011). With this network, it keeps the synchronization of each oscillator cell within a specific phase difference by using typical negative neural connection (ipsilateral) and positive connection (contralateral) to keep ipsilateral oscillation out of phase and contralateral oscillation in phase. Each cell of the four-cell network is modeled with a Hopf oscillator (Equation 1–3) which is different from the one used in Nakamura’s model (details in Discussion A.1).

(1) $$\begin{array}{rcl} {ż}_{i} & =a\left(m-z_{i}^{2}+s_{i}^{2}\right)z_{i}-\omega_{i}s_{i} & \end{array}$$

(2) $$\begin{array}{rcl} {ṡ}_{i} & =a\left(m-z_{i}^{2}+s_{i}^{2}\right)s_{i}+\omega_{i}z_{i}+\underset{j}{\sum }a_{ij}s_{j} & \end{array}$$

(3) $$\begin{array}{rcl} w_{i} & =2\times \pi \left(\frac{\omega_{\text{up}}}{1+e^{-100s_{i}}}+\frac{\omega_{\text{down}}}{1+e^{100s_{i}}}\right) & \end{array}$$

where the *z_i_* is the output of the Hopf Oscillator and *s_i_* is the internal state. *m* is the amplitude and *a* is the convergence rate. ω*_i_* is the internal weight in this coupled oscillator. It is usually set to 1. *s_j_* is the output of the other cells except cell i and α*_ij_* is the external weight (from cell j) of the four-cell network. Meanwhile, ω*_i_* also represents the frequency of this oscillator. Interestingly, by changing values of ω*_up_* and ω*_down_*, you can change the duration of increase and decrease rate of the oscillator. For example, in our work ω*_up_* = 5ω*_down_*, the oscillation increases 5 times faster than decreases. This relation is derived from the experimental data by Hallemans et al. (2006) about joint kinematic trajectories of walking children. m and a are set to be 1 and 5 in our experiment.

If we assume the motorneurons work to integrate the internal oscillation and external sensory feedback, the whole physical system including the neural controller can be expressed like this:

(4) $$ẋ=F\left(x,\tau \right)$$

where **x** denotes the state of the physical system, whose components are, for example, sensory angles of joints, and the dot (^·^) denotes the time derivative. τ denotes the control signal (torque or trajectory) from the controller, and *F*(**x**,τ) represents the vector field of the system dynamics. Then the motorneuron can be modeled by the firing neural structure (Buono and Palacios, 2004; Endo et al., 2008; Li et al., 2012), the dynamics of which can be given by:

(5) $$\begin{array}{rcll} \varsigma {ẏ}_{Ei} & =-y_{Ei}+{\text{I}}_{Ei} & & \\ \tau_{Ei} & =G_{E}\left(y_{Ei}\right) & \end{array}$$

(6) $$\begin{array}{rcll} \varsigma {ẏ}_{Fi} & =-y_{Fi}+{\text{I}}_{Fi} & & \\ \tau_{Fi} & =G_{F}\left(y_{Fi}\right) & \end{array}$$

where *y_Ei_* and *y_Fi_*, **I_Ei_** and **I_Fi_**, ζ, τ*_Ei_* and τ*_Fi_* represent the state, input, damping constants (equal to 10 in our work), and the output of ith extensor and flexor motorneuron, respectively (if no exception, all the E and F in the lowerscripts represent extensor and flexor in this article). *G_E_* and *G_F_* are both activation functions, for example the sigmoid function. The input **I***_Ei_* and **I***_Fi_* are given by:

(7) $$\begin{array}{rcl} I_{Ei} & =\underset{j}{\sum }V_{Eij}z_{j}+\underset{k}{\sum }W_{Eik}X_{Ek} & \end{array}$$

(8) $$\begin{array}{rcl} I_{Fi} & =\underset{j}{\sum }{\text{V}}_{Fij}{\text{z}}_{j}+\underset{k}{\sum }{\text{W}}_{Fik}{\text{X}}_{Fk} & \end{array}$$

where **z***_j_* is the jth output of PF layer (the four-cell network). **V***_Eij_* and **V***_Fij_* are the connection weights from PF layer to motorneuron layer. **X***_Ek_* and **X***_Fk_* are the kth sensory feedback from sensory neurons in vector **X***_E_* and **X***_F_* weighted by the connection weight **W***_Eik_* and **W***_Fik_*. Then the final output of the controller is given by:

(9) $$\tau_{i}=T_{Ei}\tau_{Ei}+T_{Fi}\tau_{Fi}+{\text{W}}_{pi}{\text{X}}_{pi}$$

where τ*_i_* is the ith output of CPGs and *T_Ei_*, *T_Fi_* are the connection weight. **X***_pi_* is the ith term in posture control vector **X***_p_* weighted by connection weight **W***_pi_*.

#### 2.1.2 Sensor neurons

The sensor neuron mechanism representing local reflex of muscles is very important for motorneurons (Latash, 2008). It has been proved to be biologically existent (Endo et al., 2008) and useful for robotic walking applications (Endo et al., 2008; Nassour et al., 2011). The general sensor neuron model is given by a sigmoid function:

(10) $$\rho_{sn}=\frac{1}{1=e^{a\left(\theta_{threshold}-\theta_{input}\right)}}$$

where ρ*_sn_* is the output of a sensor neuron. a is the sensitivity of a sensor neuron. θ*_threshold_* and θ*_input_* are the threshold and the input of a sensor neuron. The input can be raw or postprocessed sensor data and the threshold can be zero or a certain value depending on types of sensor neurons. The idea of using sensor neurons is to normalize the input of all the sensors and use them with different purposes (details see Appendix A).

According to existing robotic applications of CPGs, each CPG is used to control one joint of a robot. Each sensory connection weight (like **W***_Eik_* and **W***_Fik_*) of each CPG is determined by the corresponding joint it controls and its specific sensory input. In the layered structure implemented on the physical robot NAO (Li et al., 2012), the 4-cell network is applied to a layered CPG architecture with manually tuned weights and it represents cognitive-related prior knowledge about the fundamental properties of walking. For example, as one property this network owns, the anti-phase contralateral leg movement is useful for walking. There is evidence suggesting that this typical movement is formed over many months of early infancy before infants learn to walk (Kail and Cavanaugh, 1996; Thelen and Smith, 1996). The main focus for learning to walk is shifted from learning very basic walking prerequisites to learning how each joint is coordinated with the whole-body and adaptively reacts to environmental change. Then RL proffers a very nice blueprint.

### 2.2 NAC model

Actor-critic is a very typical but popular RL method broadly used in recent years (Kimura and Kobayashi, 1998; Sato and Ishii, 1998; Orlovskii et al., 1999; Sutton et al., 2000). In a typical implementation, an actor is a controller which emits actions or action-related control signals to a physical system. According to a certain policy, it observes the states of a physical system and determines the control signals on the basis of the states. A critic is a functional part which evaluates the states of a physical system and updates the controller and control policies. As a typical RL learning mechanism, it can be adapted by using some other updating rules. For example, the convergence of an actor-critic model based normal policy gradient approach is achieved in (Konda and Tsitsiklis, 2003) and a mathematical convergence of actor-critic is proved in (Dotan et al., 2008). The convergence of the actor-critic model with the natural policy gradient has been proved by Peters and Schaal (2008). Moreover, it has been proved to be faster than the normal “vanilla” policy gradient (Peters, 2007).

#### 2.2.1 Natural CPG-actor-critic model

**Figure 3:**
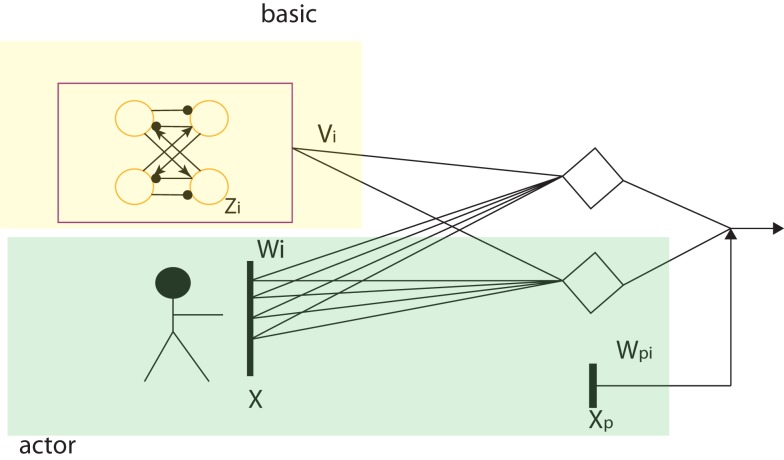
**The feed-forward two-layer neural network as the core of the CPG network**. The yellow area is the basic CPG part with fixed connection weights and the green area functions for the output integration of sensor neurons and posture control.

Natural CPG-Actor-Critic is an autonomous RL learning framework used for CPG network based on Actor-Critic learning with the natural policy gradient. It was proposed by Nakamura in 2007 and successfully implemented on Taga’s stick walker in Matlab simulation (Taga, 1998; Nakamura et al., 2007). We adopted his approach but with an entirely different CPG architecture, learning schema, and basic RL algorithm (for details, refer to discussion). Since the output of our CPG model is based on the input of PF layer and the states of sensory feedback and posture control terms, a CPG is an adaptive controller whose output is dependent on all these inputs. As a matter of fact, the layered architecture proposed in our work can be viewed as a feed-forward neural network (Figure 3) where the posture control works as a bias. As a normal gradient approach used for the feed-forward neural network, the backpropagation approach is not suitable for our work. Firstly, the backpropagation normal gradient is too slow and cannot avoid the “plateau” problem (Peters and Schaal, 2008). Secondly, it needs a lot of computation and large storage for precedent states. Therefore, the natural gradient approach is adopted as it has been proved to be more efficient than the backpropagation for feed-forward neural networks by Amari (1998) who proposed natural gradient.

Compared to Nakamura’s model, our model is naturally separated into two parts: the basic CPG and the actor part (details in Figure 3 and Discussion A.1). This is similar to Nakamura’s separation of his CPG model. The basic CPG part composed of an oscillatory network is to keep the phase relation and oscillation of the whole CPG as a core. The actor outputs the control signals based on its input. It covers two important functions of a CPG: sensory feedback fusion and posture control (Orlovskii et al., 1999). The RL updating rule can be applied to this part to change the weights, leading to involvement of the adaptive change of the CPG controller based on interaction when a robot walks. RL state space is given as **X**, a vector including all the sensory feedback and posture control terms. The action space is given by **U** which comprises all the control signals. The input and output of the CPG can be adapted to:

(11) $$\begin{array}{llll} X & \sim \left\{X_{E},X_{F},X_{p}\right\},U\sim \left\{U_{E},U_{F},U_{p}\right\} & & \\ {\text{I}}_{Ei} & ={\text{I}}_{Ei}^{basic}+{\text{I}}_{Ei}^{actor} & \end{array}$$

(12) $$\begin{array}{lll} {\text{I}}_{Fi} & ={\text{I}}_{Fi}^{basic}+{\text{I}}_{Fi}^{actor} & \end{array}$$

(13) $$\begin{array}{lll} {\text{U}}_{Ei} & ={\text{I}}_{Ei}^{\text{actor}}=\underset{k}{\sum }{\text{W}}_{Eik}{\text{X}}_{Ek} & \end{array}$$

(14) $$\begin{array}{lll} {\text{U}}_{Ei} & ={\text{I}}_{Fi}^{actor}=\underset{k}{\sum }{\text{W}}_{Fi}{\text{X}}_{Fi} & \end{array}$$

(15) $$\begin{array}{lll} U_{pi} & ={\text{W}}_{pi}{\text{X}}_{pi} & \end{array}$$

$$\begin{array}{llll} \text{W} & \sim \left\{{\text{W}}_{E},\;{\text{W}}_{F},{\text{W}}_{P}\right\} & & \end{array}$$

where ${\text{I}}_{Ei}^{basic}$ and ${\text{I}}_{Fi}^{basic}$ are the ith pair of the output of fixed basic CPG. **U***_E_* and **U***_F_* are vectors containing control signals emitted by the actor to the controller. **U***_pi_* is the ith element of a vector **U***_p_* including posture control terms. **U***_Ei_* and **U***_Fi_* are the ith terms in **U***_E_* and **U***_F_*. **W** is a vector for all the connection weights. **W***_E_*, **W***_F_*, and **W***_p_* are vectors of connection weights for sensory feedback and posture control terms. Then the RL problem could be expressed as:

(16) $$\text{U}\sim \pi \left(\text{U},\;\text{X}\right)$$

where π is the stationary policy of the RL algorithm. Clearly, all the states **X** include two parts. **X***_E_* and **X***_F_* are called observable states. **X***_p_* is called unobservable states. They are assistive states which are provided to help the robot learn a proper posture. As our idea is to learn through interaction and to sense the body through peripheral systems, there is no full observability for the whole-body states. This condition is different from Nakamura et al. (2007) application. Hence, the whole control system is regarded as a POMDP. It is indicated that the actor determines the control signals sent to CPGs according to a static policy and CPGs act with the physical system. Then the critic evaluates the locomotion under control of CPGs changed by the actor and update the policy in the actor. This is the so-called CPG-Actor-Critic. Used with the natural policy gradient, it is called natural CPG-Actor-Critic. As a proper architecture for RL learning, we need to avoid a problem of RL “the curse of dimensionality.” In order to reduce the dimensionality of the CPG controller, internal weights of the 4-cell network and **V***_Eij_*, **V***_Fij_* (1,−1) are all fixed as primitive inputs of CPGs. This is different from Nakamura et al. (2007) idea of using an internal connection from the basic CPG (). The reason for not having internal connection weights is our flexible 4-cell network has already been endowed with prior knowledge or capabilities to keep synchronization and to reshape the output of oscillators. However, this prior knowledge must be learned in Nakamura’s work. Meanwhile, using a sensory-driven CPG means there cannot be so much sensory feedback as the number of sensors on a given humanoid is always limited. Nakamura has full observability in state space of the accurate Taga walker but he only uses a subset of the available sensors. Since the aim of our work is to implement this architecture on a real humanoid to understand mechanisms of posture control and sensory feedback integration, a trial-and-error learning mechanism based on batch RL is needed (details in Discussion A.1).

#### 2.2.2 Learning algorithm

The policy gradient (PG) approach is very useful for parameterized motor modeling. Peters summarizes and compares different PG approaches, including finite difference, likelihood ratio methods, and REINFORCE (Peters, 2007). It is concluded that the aim of the gradient approach is to find the correct updating direction of policy parameters in order to maximize expected reward. Assuming the stationary policy is π^θ^(**x**, **u**) which can determine action space **u** based on state space **x** with a static distribution *d*^π^(*x*), the immediate reward is *r*(*x*, *u*), and then the expected reward *J*(θ) can be written as:

(17) $$J\left(\theta \right)=\int_{\text{x}}d^{\pi }\left(\text{x}\right)\int_{\text{u}}\pi^{\theta }\left(\text{u}|\text{x}\right)r\left(\text{x},\text{u}\right)d\text{x}d\text{u}$$

where the policy π^θ^(**x**, **u**) is derivable at the policy parameters θ, namely ▽_θ_π^θ^ exists. For maximizing expected reward *J*(θ) with respect to θ, policy gradient will find the steepest increase direction ▽_θ_*J* = *J*(θ + ▽θ) − *J*(θ) to update the search policy π^θ^(**x**, **u**) until it converges. For this purpose, the update rule of the policy gradient can be expressed as:

(18) $$\theta_{n+1}=\theta_{n}+\alpha \nabla_{\theta }J{|}_{\theta =\theta_{n}}$$

where n represents the nth step of update and α is the learning rate (equal to 0.01). If we directly take the 1st derivative of *J*(θ) with respect to θ, the gradient is given by:

(19) $$\begin{array}{lll} \nabla_{\theta }J\left(\theta \right) & =\int_{\text{x}}d^{\pi }\left(\text{x}\right)\int_{\text{u}}\nabla_{\theta }\pi^{\theta }\left(\text{u}|\text{x}\right)r\left(\text{x},\text{u}\right)d\text{x}d\text{u} & \end{array}$$

(20) $$\begin{array}{lll} & =\int_{\text{x}}d^{\pi }\left(\text{x}\right)\int_{\text{u}}\pi^{\theta }\left(\text{u}|\text{x}\right)\nabla_{\theta }\log\left(\pi^{\theta }\left(\text{u}|\text{x}\right)\right)r\left(\text{x},\text{u}\right)d\text{x}d\text{u} & \end{array}$$

where ▽_θ_ is the 1st derivative. This is the so-called normal gradient. If we use this gradient to update the policy, it is very slow to find the best policy for the maximization of expected reward. Therefore, the steepest gradient (natural policy gradient) is applied to our model. The adaptation of Equation 20 is at the core of the natural PG method. According to Peters’ (2007) proof, the natural gradient is given by:

(21) $$\begin{array}{lll} \theta_{\text{n}+1} & =\theta_{\text{n}}+\alpha F_{\theta }^{-1}\nabla_{\theta }J{|}_{\theta =\theta_{\text{n}}} & \end{array}$$

(22) $$\begin{array}{lll} F_{\theta } & =\int_{T}\pi^{\theta }\nabla_{\theta }\log\pi^{\theta }\nabla_{\theta }\log\pi^{\theta }d\theta & \end{array}$$

where F is the Fisher Matrix (FM). Multiplied by FM, the normal policy gradient is changed to the steepest one (here, all the **x**,**u** are neglected for simplification reason). On the basis of policy gradient theorem (Peters, 2007), the PG could also be modified to:

(23) $$\nabla_{\theta }J\left(\theta \right)=\int_{\text{x}}d^{\pi }\left(\text{x}\right)\int_{\text{u}}\nabla_{\theta }\pi^{\theta }\left(\text{u}|\text{x}\right)\left(Q^{\pi }\left(\text{x},\text{u}\right)-b\left(\text{x}\right)\right)d\text{x}d\text{u}$$

where *Q*(*x*,*u*) is the action-state function and *b*(*x*) is a baseline which is a regularized term used to avoid large variance of gradient. With the theory of compatible function approximation, it is possible to apply basis functions ▽_θ_*log*^T^(π^θ^(**u**|**x**)) to linearly approximate *Q*^π^(**x**, **u**) − *b*(**x**), then the above Equation 23 is adapted to:

(24) $$\begin{array}{llll} \nabla_{\theta }J\left(\theta \right) & =\int_{\text{x}}d^{\pi }\left(\text{x}\right)\int_{\text{x}}\pi^{\theta }\left(\text{u}|\text{x}\right)\nabla_{\theta }\log\left(\pi^{\theta }\left(\text{u}|\text{x}\right)\right) & & \\ & \;\times \nabla_{\theta }\log^{T}\left(\pi^{\theta }\left(\text{u}|\text{x}\right)\right)wd\text{x}d\text{u}=F_{\theta }w & \end{array}$$

where **w** is a weight vector of the linear approximation. Then clearly, by replacing ▽_θJ_(θ) in (21) with (24), the natural PG becomes:

(25) $$\theta_{\text{n + 1}}=\theta_{\text{n}}+\alpha \text{w}$$

The RL problem is transitioned from searching the steepest policy gradient to a normal regression problem about finding the best approximation of *Q*^π^(**x**, **u**) − *b*(**x**) with basis functions. Because $Q^{\pi }\left(\text{x},\text{u}\right)=b\left(\text{x}\right)+\log\left(\pi^{\theta }\left(\text{u}|\text{x}\right)\right)\text{w}$ and $Q^{\pi }\left(\text{x},\text{u}\right)=r\left(\text{x},\text{u}\right)+\lambda \int_{\text{x’}}p\left(x^{\prime }|x,u\right)V\left(x^{\prime }\right)dx^{\prime }$ (where λ is the discounting factor, **x′** is the next state, *p*(**x′**|**x**,**u**) is the probability of state transition.), assume the value function is *V* (**x**) = *b*(**x**) and can be approximated by ψ*^T^*(**x**)**v** (where **v** is the weight vector and ψ is the vector of basis function related to the value function; Baird, 1994). Therefore, the approximation can be re-written:

(26) $$\begin{array}{llll} \log^{T}\left(\pi^{\theta }\left({\text{u}}_{t}|{\text{x}}_{t}\right)\right)\text{w}+\psi^{T}\left({\text{x}}_{t}\right)\text{v} & =\text{r}\left({\text{x}}_{t},{\text{u}}_{t}\right)+\lambda \psi^{T}\left({\text{x}}_{t+1}\right)\text{v} & & \\ & \;+\in \left({\text{x}}_{t},{\text{x}}_{t+1},{\text{u}}_{t}\right) & \end{array}$$

This is the equation for *LSTD*-*Q*(λ) at time t. Then for the episodic learning, by summing up equation (26) with *t* = 1,2…*H*, it is given by:

(27) $$\frac{1}{H}\overset{H}{\underset{t=1}{\sum }}\log^{T}\left(\pi^{\theta }\left({\text{u}}_{t}|{\text{x}}_{t}\right)\right)\text{w}\text{ + J}=\frac{1}{H}\overset{H}{\underset{t=1}{\sum }}r\left({\text{x}}_{t},{\text{u}}_{t}\right)$$

where *J* is the value-function related term considered as a constant baseline. By means of the least square learning rule, the natural PG **w** can be obtained for each episode:

$$\left(\begin{array}{c} \text{w}\\ J \end{array}\right)={\left(\phi \phi^{T}\right)}^{-1}\phi R.$$

(28) $$\phi_{t}=\left[\frac{1}{H}\sum_{t=1}^{H}\log^{T}\left({\text{π}}^{\theta }\left({\text{u}}_{t}|{\text{x}}_{t}\right)\right)\text{w},1\right]$$

(29) $$R=\frac{1}{H}\sum_{t=1}^{H}r({\text{x}}_{\text{t}},\;{\text{u}}_{\text{t}})$$

In our work, we use a monte-carlo like approach called episodic NAC (eNAC) (Peters, 2007) to make the robot repeat the walking episodes until it achieves final optimal performance. The eNAC is shown in Schema 1 with pseudocode.

Schema 1 *Repeat*: n = 1,2 … M trials

*input*: policy parameterization $\theta^{n}$

π(**U**|**X**) determines **U***_p_* before starting each trial

*Start the trial*: obtain **X**_0:*H*_, left **U**_0:*H*_,*r*_0:*H*_ for each trial from π (**U**|**X**)

Obtain the sufficient statistics

policy derivatives: $\phi_{k}=\nabla_{\theta }\log{\text{π}}_{\theta }(U_{t}|X_{t})$

Fisher matrix $F_{\theta }=\langle \left(\sum_{k=0}^{H}\phi_{k}\right){\left(\sum_{l=0}^{H}\phi_{l}\right)}^{T}\rangle$

Vanilla gradient $g=\langle \left(\sum_{k=0}^{H}\phi_{k}\right)\left(\sum_{l=0}^{H}\alpha_{l}r_{l}\right)\rangle$

Eligibility $\psi =\langle \left(\sum_{k=0}^{H}\phi_{k}\right)\rangle$

General reward $\bar{r}=\left\langle \left(\sum_{l=0}^{H}\alpha_{l}r_{l}\right)\right\rangle ,$ where α^l^ is the discount factor

Obtain natural gradient by computing

baseline $b=Q\left(\bar{r}-\psi^{T}F_{\theta }^{-1}g\right)$

with $Q=M^{-1}\left(1+{\text{ψ}}^{T}{\left(MF_{\theta }-{\text{ψψ}}^{T}\right)}^{-1}\psi \right)$ *When updating rule is satisfied:*

$\theta_{n+1}=\theta_{n}+\alpha g$

until the convergence of algorithm

where 〈 · 〉 means sum-up of all the previous values and current values.

### 2.3 Experimental settings

There are 2 main experiments presented in this article. The first one is to indicate that the proposed learning architecture can assist the robot learning to walk from the initial standing posture. The aim of this experiment is to prove the robot can adjust its posture and integrate sensory feedback simultaneously in the process of learning. The second experiment is to change the plane on which the robot stands to different angles to see how the learning architecture adaptively seeks out proper postures and walking gaits. By changing angles from −5° to +5°, this experiment also shows the relation between slope angles and posture change under the influence of gravity alternation.

#### 2.3.1 Robotic platform and the neural controller

**Figure 4:**
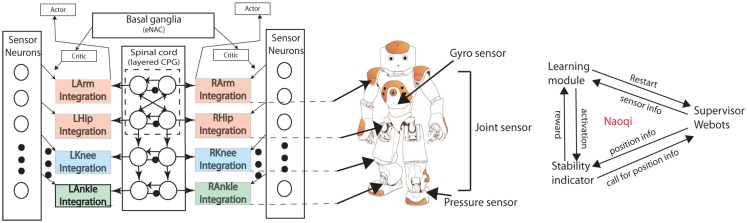
**Left: The complete architecture of cpg-actor-critic network**. The integration with specific joint names represents the functions of motorneurons for each joint. The architecture abstractly represents the neurophysiological structure of the brainstem where the basal ganglia is in charge of RL, and the spinal cord is where the CPGs are located and motorneurons. Each layer of the three-layered architecture corresponds to different parts. The actor-critic learning mechanism works with basal ganglia as a RL functionality. Sensor neurons are different types of neurons which get information from different sensors of the robot (middle). Middle: The NAO robot. Arrows indicate the connection between the controller and the robot. Right: the software architecture. Naoqi is working as a middleware to handle the communication of three modules. The communicative information between every two modules are listed above the arrow.

Figure 4 shows the robot and the neural network used to implement learning. We use the popular commercialized robot NAO. The advantages of using the NAO robot are summarized as: (1) There are locomotion-relevant sensors mounted on the NAO robot, such as gyro sensors which can detect acceleration of the body center in 3D space, joint sensors which can measure angle values, and foot pressure sensors which can sense ground contact of feet. All these sensors are useful for learning a proper walking gait. (2) Nao has a good firmware called Naoqi which is convenient for users to program and organize modules working together.

The layered CPG network (Figure 4 left) is used to control the NAO robot. Each output sends out position trajectories to each corresponding joint of NAO. Simultaneously, all the CPG neurons receive inputs from different kinds of sensor neurons based on the concept of sensor-driven CPG. There are three main sensor neurons with similar sigmoid form (refer to Appendix A): Proprioceptive (PP) sensor neurons for hips (joint sensors), anterior extremity (AE) sensor neurons for knees (joint sensors), and exteroceptive (ET) ankle sensor neurons (mixture of gyro sensors and pressure sensors). The motion of pitch direction is controlled by the CPG neural network while the roll motion (hips and ankles) is sensor-driven by the pitch motion (hips and ankles), respectively (Li et al., 2012; Appendix A).

#### 2.3.2 Software

In this work, we use a simulated environment in the Webots simulator. Webots is an ODE (Open Dynamics Engine) based simulator in which users can not only simulate physics close to the real world but also move robots or objects and even change the environment. This is why there is a typical feature of Webots for simulating batch learning processes (Michel, 2004).

There are three main modules working together in the Naoqi of Webots. The supervisor module is in charge of restarting the simulation every episode by putting the robot in the initial position, changing the angle of the ground, measuring the distance the robot walks for each episode. The learning module is the main process where the CPG architecture and the learning algorithm are implemented. The stability indicator is a module working only for obtaining necessary sensory information from the supervisor module and the robot as well as calculating the immediate reward. It is an implementation of a basal ganglia like function. It sends a reward to the main process when activated by the learning module (Figure 4).

## 3 Results

### 3.1 Experiment 1: walking on the flat ground

#### 3.1.1 Prerequisites

In this experiment, the robot starts to walk from the same initial default standing posture and repeats the episode which lasts about 30 s until the algorithm converges. At the beginning of each episode, the policy gives two posture control signals for the knee and ankle parts as the posture change is very sensitive and should be explored as a basis for motion. Within each episode, the policy gives the other control signals related to sensory feedback every 1.5 ms. The policy used for balancing exploration and exploitation is given:

$$\begin{array}{llll} \pi_{\theta }\left(\text{U},\text{X}\right) & =N\left(\text{U},\bar{\text{U},}\sigma \right) & & \\ & =\frac{2\pi }{\sigma }\exp\left(\frac{\left(\text{U}-Ū\right){\left(\text{U}-Ū\right)}^{T}}{\sigma^{2}}\right) & & \end{array}$$

where **U** is the output vector of the policy and $\bar{\text{U}}$ is the input vector based on state space **X**. σ is the exploration rate which determines the variance of **U** from $\bar{\text{U}}$. The value of σ cannot be so big (>0.1) that the system involves a lot of noise and it cannot be too small (<0.01) as the system will become very insensitive and diverges. In this experiment, for the posture control part **U***_p_*, σ = 0.05. Otherwise σ = 0.02. As 0.02 is too small for the posture terms, a slightly bigger exploration rate is adopted. After having the continuous control signals sent to each joint, the robot needs to have the capability of evaluating different appearing walking gaits. The immediate fitness of a walking gait is acquired every 1.5 ms via the reward function which indicates the gait robustness, also called stability indicator. The stability of a walking gait should be considered in two directions: vertically, the SI is able to detect falling; horizontally, SI also considers the distance the robot moves. In this way, SI reflects a trade-off between vertical and horizontal stability. Thus, the SI is given:

(30) $$r=r_{height}+r_{acc}+r_{distance}$$

where $r_{height}=e^{25\left(H-H_{inil}\right)}\text{,}$ *H* is the height of gravity center and the NAO robot can detect the height based on the gyro sensor. *H_init_* is the height of gravity center of the initial standing posture. Thus, this equation detects a dynamic change of height of the body when the robot is walking. When the robot falls, it is close to 0. $r_{acc}=2\cos\left(\frac{accX}{10}\right)+2\cos\left(\frac{accY}{22}\right),$ if $\left|accX\right|<25$ and $\left|accY\right|<50.$ Otherwise, the robot is stopped and the episode is restarted. *accX* and *accY* are the acceleration of the robot’s X axis (Pitch) and Y axis (Roll) of gravity center detected from the gyro sensor. For both directions, the gyro sensor is able to detect the acceleration from −70 to 70 which corresponds to −9.8 to +9.8 m/s^2^. This part is implemented based on the inspiration of a vestibular system in the inner-ear mechanism for keeping body balance. It senses “falling” of the body by detecting the accelerations in 3D space (Thomas et al., 2009). Here, as we aim to study walking on the ground, the perpendicular acceleration is ignored. Twenty-five and 50 are the boundary values for the robot to fall. The even *cos* function is used to indicate this oscillatory motion of the walking in negative and positive directions of each axis. *r_distance_* = 2S and *S* is the walking distance detected by the supervisor module in Webots.

After each episode, two kinds of average reward are acquired. One is the average reward (AR) for each episode equal to $\overset{H}{\underset{l=0}{\sum }}a_{l}r_{l}$ and the other is the general average reward (GAR) equal to $\frac{\left\langle \overset{H}{\underset{l=0}{\sum }}a_{l}r_{l}\right\rangle }{M}.$ If *AR* > *GAR*, the updating rule is satisfied. Otherwise, the episode is regarded as a failure. The algorithm converges when the learning process cannot find any episode which can satisfy the update rule.

#### 3.1.2 Experiment 1 results

**Figure 5:**
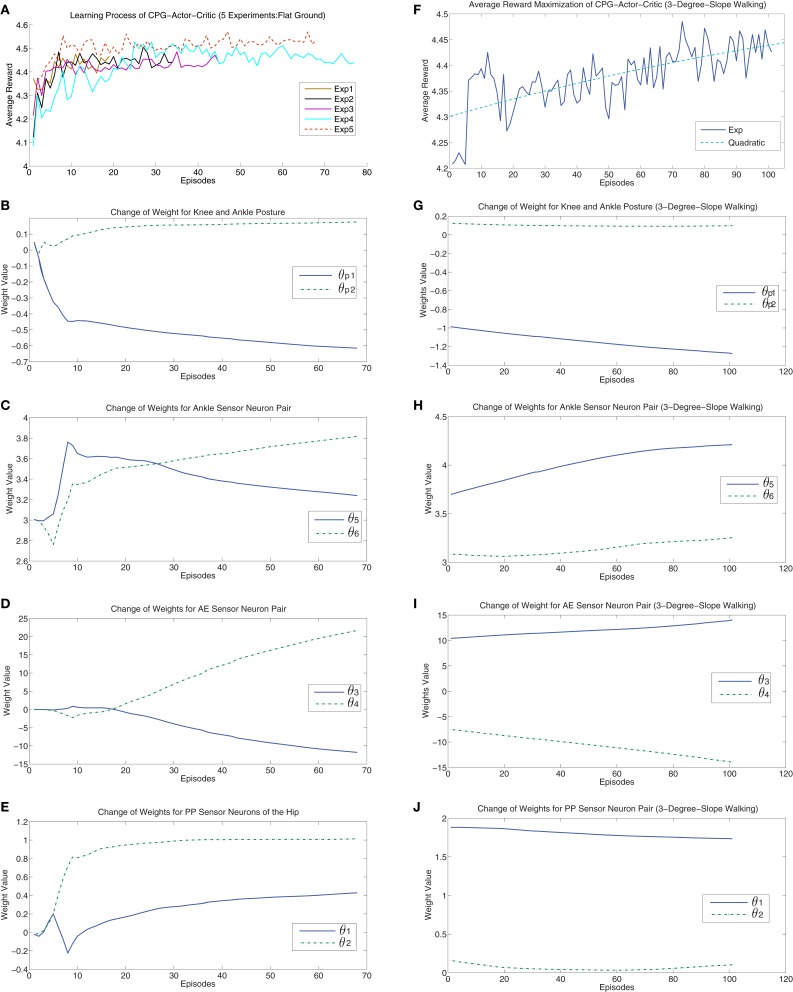
**Left column: The results of the runs with top-five reward on flat ground**. **(A)** shows the maximization of average reward for the five runs. **(B–E)** show the results of the run with highest average reward (Exp 5) regarding how connection weights are updated in each CPG by learning process with respect to the contributions of each term respectively. Right colunm: The results of a run on 3° critical slope. **(F)** shows the “struggling” maximization of expected reward. The green dash line shows a quadratic fitting of the increasing learning curve. **(G–J)** show how connection weights of CPGs are adaptively updated on the critical slope. For details of explanation, please refer to main text. All the “Episodes” mean updating episodes which exclude the episodes unable to satisfy updating rule.

For each experiment, the algorithm starts with initialized θ = 0 except that θ_5_ = θ_6_ = 3 as 3 is the weight value making ankle sensor neurons sensitive to external disturbance. 10 independent runs (different random seeds) were evaluated and 5 “good” results with top-five average reward are chosen for visualization in Figure 5 (left column). We chose the one with highest average reward (run 5) to show how cpg-actor-critic finds the optimal learning gradient. Actually, the key feature of cpg-actor-critic is that it can find the best update directions of parameters quickly via balancing the exploration and exploitation. It is clearly observed that in the very first 10 episodes, the update directions of all the parameters are not stable, even opposite of right directions. However, after 10 update episodes, cpg-actor-critic can quickly find good and smooth update paths. Interestingly, Figures 5B–E shows the convergence of posture related parameters. In Figure 5B, θ*_p_1* and θ*_p_2* shows the posture change of the knee and the ankle. The knee posture is extending (θ*_p_1* turns negative) a lot to move the center of gravity toward the middle while the ankle position is only slightly changed to keep the balance with the knee posture. Meanwhile, θ_2_ is increasing to 1 in order to limit the extension of the hip part and strengthen the flexion of the hip motion. The posture change of a chained-up three joints (ankle, knee, and hip) drives the robot to walk more robustly and for a longer distance. The final convergence of proper posture for walking is a consequence of the interaction of the morphology of NAO, the neural controller and the sensory feedback. For example, it is logical that NAO’s ankle cannot be changed a lot as it is disproportionately big. The cpg-actor-critic realizes this obviously by the slight adjustment of the ankle posture with interaction.

As for the connection weights of AE and ankle sensor neurons, they only show the curves without flat convergence. The reason is that, in eNAC, the *Q* function is actually theoretically approximated by a linear combination of basis functions. However, practically it is only possible to averagely approximate without exact accurate convergence. This is also the reason we need to set up a specific convergence rule.

Finally, a specific walking gait is converged to by the interactive learning process and parameters are converged to θ = [0.4290, 1.0131, −11.7874, 21.6984, 3.2394, 3.8179, −0.6147, 0.1758, −12.8070].

### 3.2 Experiment 2: Walking on the slope

#### 3.2.1 Prerequisites

The aim of experiment 2 is to test if the learning architecture can still function when there is different non-linear influence of the gravity for walking up and down the slope. Meanwhile, it is interesting to observe how the robot adaptively reacts to environmental change by achieving a trade-off between adaptation and learning. Finally, a conclusive relation between adaptive adjustment of CPG parameters and slope is explained.

In this experiment, we fully adopt the architecture in Figure 4. Since results in experiment 1 do not show any qualitative difference of walking gaits, each run in experiment 2 uses the parameter set developed in an arbitrarily selected good solution from experiment 1. The NAO, in each evaluation, is thus able to walk on a flat slope before attempting an upward or downward slope, depending on the condition. The good solution obtained for flat-ground walking consists of the following parameter set: θ = [1.3391, 0.4717, 3.1593, −0.6291, 3.4483, 3.1432, −0.6640, 0.2293, 0.4365] used as the set of values at the start of each experiment 2 run. In each experiment 2 run, the architecture is tested to learn to walk on the slopes from −0.08–0.08 rad (about −5–5°) by changing 0.01 rad each test. For each slope, there are 5 runs carried out for each condition where the aforementioned angles (8 in total) are gradually varied (get steeper) over the course of each simulation. Therefore, there is a total of 8 * 5 upslope and 8 * 5 downslope angles from which data points are derived (see Figure 7).

#### 3.2.2 Experiment 2 results

**Figure 6:**
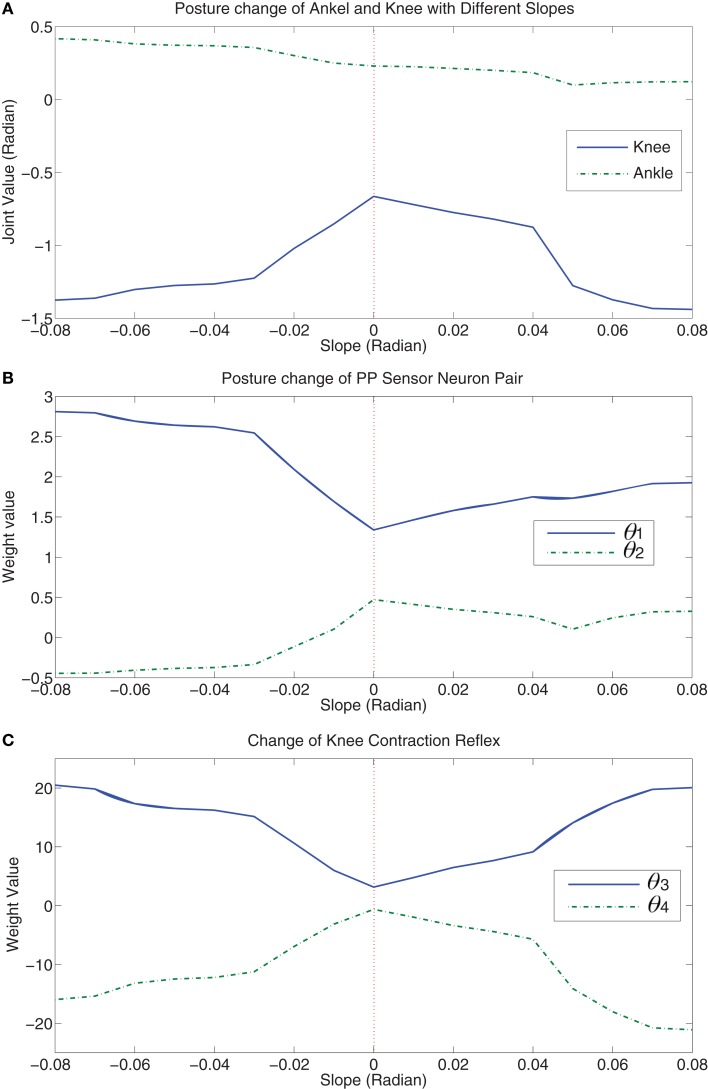

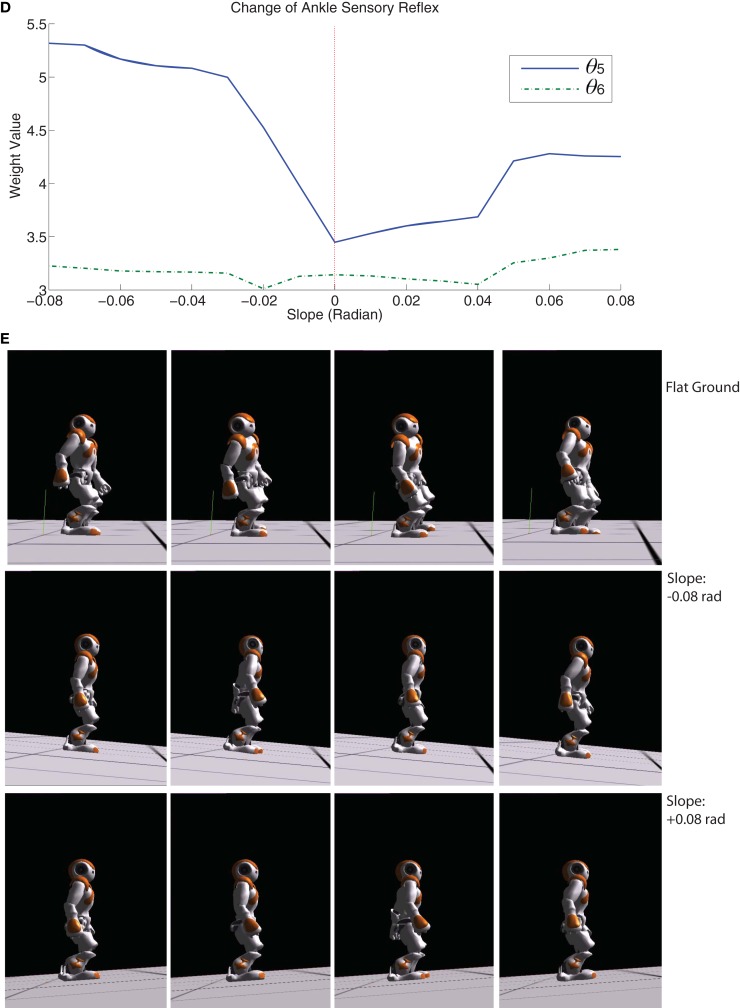
**(A)** Posture change of ankle and knee joint with respect to slope (−0.08∼0.08). **(B)** shows how the hip joint is adjusted to adapt to slope changes. **(C,D)** show how the knee and ankle reflex change with respect to slope based on the strength of sensory feedback. **(E)** shows the different walking gaits on flat ground and slope (−0.08 and +0.08 rad). Please refer to video (Cai, 2013).

Walking up and down the slope are two different cases with distinct gravitational effects. Figure 6 shows how the walking posture and sensory feedback are autonomously changed by learning in those two situations (average data). From negative slope to positive slope, the change of gravity exerted on the robot is a non-linear alternation. So the posture change is required to cancel the influence of gravity in the moving direction (upslope and downslope: extra negative and positive force respectively). If we assume the slope is β, then the gravity exerted in the walking direction is given by *f* = *mgsin*β, where m is the mass of the robot and g is the gravity constant. Therefore, Figure 6A shows a non-linear change of knee posture. When the robot walks up the slope, the gravity is a resistance force. When β is very small, *mgsin*β ≈ *mg*β shows a linear-like relation in which there is only small error. When the errors are accumulated until the resistance force *f* starts to prevent the robot moving forward, then the non-linear change has to be canceled. This is why there is an abrupt change when the robot walks up on the 3° slope (0.05 rad) which is called “critical” slope. Then when the slope is slightly steeper than 0.05 rad, Figure 6A shows a new linear change of the knee posture. The same phenomenon happens to be the case that the robot walks down the slope (slope −0.04 is a turning point). Figures 5E–J show the updating of parameters for the “critical” slope. It is clearly visualized that a smooth parameter adjustment of the 3°-slope walking is achieved after the optimal update direction has been found by the learning process of previous slope walking. Interestingly, the posture alternation of the ankle part shows a nearly perfect linear change with respect to alternative slopes. The possible reason may be led by the sensory feedback (refer to the terms **X***_E_3* and **X***_F_3* in Appendix A) adaptively changing the ankle posture according to the inclination angle (detected by the gyro sensor) of the robot. This sensory feedback shows the natural adaptation of the CPG architecture which compensates accumulated errors (a non-linear weight change of ankle sensor neurons compensates the gravity in Figure 6D). As the key to maintaining stable walking is how to hold up the walking posture as upright as possible, the change of one joint in a kinematic chain of the leg leads to a posture alternation in other joints. Therefore, when the slope is turned from −0.08 to 0.08 rad, with nearly symmetric knee posture change and decreasing ankle change, the hip motion naturally flexes more on the upslope (pushing the body upward) and extends more (flexes less) on the downslope (using the gravity of the body). In Figure 6B, the alternation of θ_1_ of downslope walking is larger than that of upslope walking indicates that the robot needs more hip flexion for walking on the upslope than the downslope. Figures 6A,B insinuates a maintenance of upright walking posture on different slopes.

As for the sensory feedback integration, the knee reflex has a symmetric tendency of upslope and downslope walking (Figure 6C). The ankle reflex changes non-linearly to compensate the effect of non-linear gravity change on the ankle joint (Figure 6D). Therefore, with an appropriate posture control and decent sensory information, the robot converges to different walking gaits on flat ground, upslope, and downslope (Figure 6E). The main difference between the gaits on flat ground and slope except posture is that the amplitude of roll motion is automatically reduced in slope walking in which case that slope walking needs more prudent gaits.

#### 3.2.3 Data analysis

**Figure 7:**
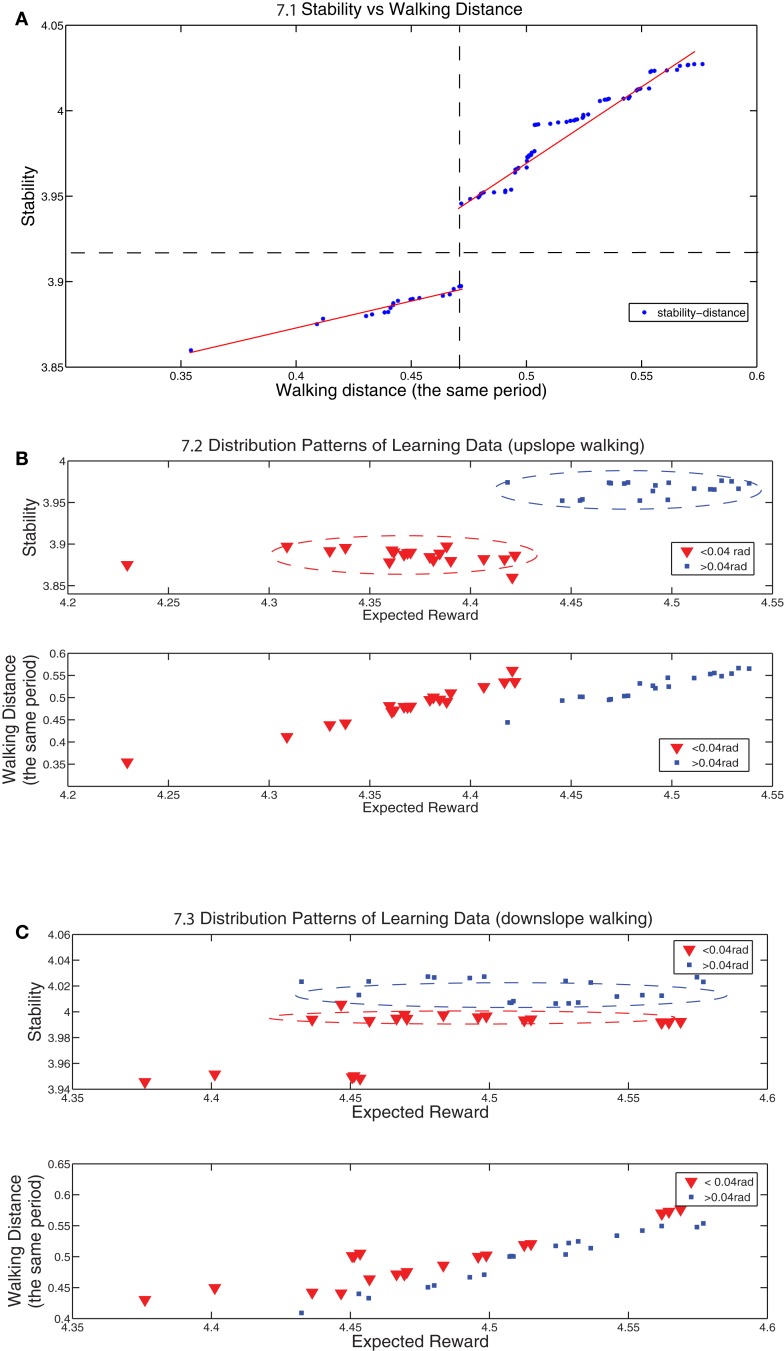
**(A) shows distribution points of stability vs walking distance for both upslope and downslope walking (80 data points)**. The dashed lines split the region into two regions: the left-upper cluster represents the results whose reward are above 4.4 and the right-down cluster represents the results whose reward are between 4.3 and 4.4 except one dot whose reward is below 4.3. Both of these two clusters are distributed around two hand-drawn lines. **(B,C)** show the distribution points of stability vs reward and walking distance vs reward for upslope and downslope walking respectively. The red-triangle dots represent the results for the cases in which |*slope*| < 0.04 *rad* and the blue-plus dots represent the results for |*slope*| > 0.04 *rad*. Note that the walking distance is measured always for the same period and it also reflects the speed of walking.

The distribution of experimental data is shown in Table 1. Based on the reward, the data is categorized into three groups in accordance with Figure 7A and the number of results are grouped into these three categories. It is shown both in Figure 7A and Table 1 that most of learning results converge to the reward above 4.3 and 81.3% converged walking gaits are obtained with the reward above 4.4 which are dubbed as good results. In Figure 7A, the data shows two linearly increasing relations between the stability and walking distance, proving that the RL learning tries to optimize both of two key factors important for a good walking gait (According to Equation 30, the reward function is equal to the sum of stability and walking distance). Figure 7B indicates an interesting boost for the stability at the “critical” slope (0.04 rad) observed in the last section. Two stability clusters are observed in Figure 7B (upper). The learning algorithm maintains the stability on two levels separated by the “critical” slope and tries to imporve the walking distance as much as possible (Figure 7B (down)). Similarly, the same boost occurs for downslope walking with the separation of |*slope*| = 0.04. However, the stability of downslope walking is more than upslope walking as an acceleration in the forwarding direction is demanded in order to walk upward (In our work, stability is negatively proportional to the acceleration of the robot’s pitch and roll directions). Therefore, with less force exerted on the body (less acceleration) and the same walking distance, downslope walking is easier compared to upslope walking in our experiments.

**Table 1:** **The Distribution of Experimental Data**.

Reward	Upslope walking	Downslope walking
<4.3	1	0
4.3–4.4	9	5
>4.4	30	35

### 3.3 Conclusion

With the two experiments, the natural cpg-actor-critic architecture successfully learns different gaits through interaction according to environmental change. It also learns the correlation of posture changes amongst ankles, knees, and hips based on the NAO robot’s morphology and the adaptability of neural controller. Meanwhile, it also achieves the implementation of CPG adjusting posture and integrating sensory feedback at the same time.

## 4 Discussion

### 4.1 Comparison of our work with related work

#### 4.1.1 Comparison to Nakamura’s model

In order to explain the features of the proposed natural cpg-actor-critic in this article, the comparison of our model to Nakamura’s is helpful to generally comprehend this complicated architecture.

##### 4.1.1.1 Similarity

Based on the NAC, Nakamura’s model and ours are both natural cpg-actor-critic architecture for learning walking gaits in different environments. The two architectures both layer into basic connections and training connections. The advantage of layering is to reduce the dimensionality of parameter space to avoid the typical problem for reinforcement learning (RL), curse of dimensionality.

##### 4.1.1.2 Differences

1. The use of a robot platform is different. Apparently, Nakamura’s model only works on Taga’s stick walker in Matlab. The work shown in this article covers an implementation on a real robot in a simulated physical world. The interaction of morphology, environment, and sensory feedback is closer to the physical world. This is the first implementation of natural cpg-actor-critic on a real robotic platform according to the authors’ knowledge. The NAO robot is a robot which moves in 3D space and is more complicated than the 2D stick walker.
2. The type and use of CPG are both different. Nakamura’s model is based on Matsuoka oscillators while Hopf oscillators are used in our work. The main difference of these two oscillators is that a Hopf oscillator can change its pattern simply by adjusting ω*_i_* to preserve the basic characteristics (longer descending phase than ascending phase and anti-phase of the two legs) of walking behaviors but a Matsuoka oscillator cannot (Righetti, 2008). In this article, our CPG architecture is inspired not only by the layered biological structure but also by a sensor-driven mechanism. Sensor neurons are very useful to endow CPGs with preliminary adaptation.
3. The learning mechanism is distinct. As abovementioned, our model reduces more computation load and dimensions by grounding basic properties of walking in the PF layer. On the other hand, by using baseline *b* in eNAC is helpful in stabilizing the RL algorithm. This is why our model learns much faster and is more stable (not easily get diverged) than Nakamura’s.

Generally speaking, the two natural cpg-actor-critic models are distinctly implemented in different bodies in heterogeneous physical worlds with dissimilar use of CPGs.

#### 4.1.2 Features of our work

Except for the characteristics compared to Nakamura’s model, our work also generally presents several novel features/perspectives compared to related work (Matsubara et al., 2006; Manoonpong et al., 2007; Endo et al., 2008; Nassour et al., 2013):

1. Morphology logic: the traditional inverse kinematics (IK) model is not used in our model. IK provides a mapping from cartesian space to joint space as long as a trajectory of the end-effector is known. However, walking does not necessarily need IK (McGeer, 1990; Manoonpong et al., 2007; Nassour et al., 2013). Even though IK is coined as a morphological logic for a rigid-body robot (Pfeifer and Bongard, 2006), our work may imply that IK is not the only logic and the interactive memory (Eligibility ψ for natural gradient) can also form a logic to help robot adjust the body posture adapting to environmental change. In Endo et al’s. (2008) work, a walking CPG model (only on flat ground) based on IK is presented and the trajectory the foot follows is presumed to be a predefined ellipsoidal path. In our work, the posture is adjusted according to the gradient update interactively focusing on body stability and walking distance instead of recalculating the foot trajectory on different terrains (slope or flat ground). In Nassour et al’s. (2013) work, the posture control is only implemented on the ankle part and it is manually tuned. However, our CPG model not only learns the weights of posture control term for the ankle part but also form an adaptive morphological logic by adapting posture alternation to different slopes. As for the work in Manoonpong et al. (2007); Matsubara et al. (2006), a simplified leggy walker without ankle joints is utilized, which seems to make it easier for the robot to walk.
  In a nutshell, in most of the work, an initial posture is manually chosen to be a basis/center which CPGs oscillate around but the evaluation of the posture remains unknown. In our work, we involve a posture control mechanism so that the posture is also adaptively changable to alternative terrains on the basis of past experience.
2. Learning mechanism: our work is the first implementation of natural cpg-actor-critic on a complete humanoid. “Natural” means the gradient approach applied in our model is the steepest and exploration-efficient in light of using natural gradient (Peters and Schaal, 2008). The RL learning presented in the work (Endo et al., 2008; Matsubara et al., 2006) is based on non-natural gradient which may not effectively avoid the “plateau” problem that the small gradient update causes learning to be stuck in a local optima without final convergence. On the other hand, in terms of dimensions of parameter space, our model has the ability to learn by adapting 9 parameters together. In Nassour et al’s. (2013) work, there are only two parameters tuned and all the other connection weights are manually defined, including the posture change parameters for ankle parts. In Endo et al’s. (2008) work, it is based on a speed-up normal gradient with three parameters to optimize. Therefore, our model seems to be able to work in a relatively high-dimensional parameter space.

However, there are still unsolved problems remaining in our work and they are summarized as follows:

1. Lack of memory: In our work, we demonstrate a CPG architecture leading the humanoid to learn to walk on different slopes. However, we acquire different adapted values of parameters with the same configuration of the parameter set. In order to adapt to the environmental change, this architecture needs spatio-temporal memory to memorize the relation between learned parameters and environmental variables. For example, in our work, contextual variables (the angle of the body) can be detected by gyro sensor. With the spatio-temporal memory, the robot can perform adaptive walking without learning when encountering the contextual changes it has experienced and learned before. The contextual transition may be solved by context-related transition based on bifurcations (Asa et al., 2009) or a context-switching mechanism with topological map (Caluwaerts et al., 2012).
2. Transferability: Even though most of related work demonstrates the results in a simulated robot (Matsubara et al., 2006; Manoonpong et al., 2007; Endo et al., 2008), whether our work is transferable to the physical robot still remains uncertain. In future work, we have to test different results on the physical robot.

#### 4.1.3 Insights into RL approach selection

For the POMDP we concern in this article, function approximation is a very useful solution for solving problems in continuous action space (Orlovskii et al., 1999). Discretizing the state space with feature input of an agent is commonly used approach in actor-critic to representing the states of an agent under the condition that the state space is infinitely large (Orlovskii et al., 1999). Therefore, the value function can be approximated in a lot of ways. For example, it could be approximated based on state predictors (Doya et al., 2002; Gianluca, 2002; Khamassi et al., 2006), artificial neural network (ANN) (van Hasselt, 2011; Farkaš et al., 2012), and basis functions (Doya, 2000b; Peters and Schaal, 2006; Nakamura et al., 2007; van Hasselt and Wiering, 2007). Regarding to the approximation based on state predictors, they mainly work for multi-model model dependent applications so it is not easy to compare the performance among them. It seems Cacla proposed by Hasselt can be adapted with ANN very easily for both actor and critic for the value-function approximation and action selection (van Hasselt, 2011). In our work, we mainly use episodic NAC to achieve steepest policy update. However, Hasselt et al compare NAC and Calca on cart-pole tasks, finding that Calca outperforms NAC (Orlovskii et al., 1999). The main difference between NAC and Calca is that the former optimizes the policy which maps state space to action space and the latter can search optimal solutions in action space directly. This is why Calca can update the action and approximate the value function separately with two sets of parameters and the action parameters are only updated with positive temporal difference (TD) (van Hasselt and Wiering, 2007). Normal NAC has to update also with negative-TD causing the action space to jump into an unknow space which may distablize and fail NAC. Inspired from Calca, in our work, we use the positive-TD update rule (*AR* > *GAR*) to avoid the suffering of negative-TD update for NAC. With initial trials for using Calca on cpg-actor-critic, it seems Calca cannot converge even after 300 episodes as it updates slowly.

### 4.2 Dynamic systems approach

Walking, in dynamic systems theory (DST), is regarded as a flexible limit-cycle behavior. Learning to walk entails finding out a proper limit cycle of the body motion in a certain environment through interaction. The cpg-actor-critic, as the architecture based on this theory, also covers a lot of aspects of the dynamic systems approach. According to Thelen, a dynamic system could be viewed as an equation *q* = *N*(*q*, *parameters*, *noise*) where q is a vector representing all the subcomponents or states of the system and parameters are key factors to which the collective converged behavior is sensitive and that shift the system through different states. N is a non-linear function which determines q which reflects an attractor (Thelen and Smith, 1996). Similarly, the cpg-actor-critic could be written as *cpg* = *N*(*cpgstates*, θ, *noise*) where *cpg* is the vector of all the output of CPGs, cpg states are **X** and θ is a vector containing policy parameters. N represents the RL functionality which can find an attractor of CPGs. The noise is compressed with proper exploration rate of policies. The whole system is wrapped for a non-linear process of searching for attractors. In a dynamic system, *q* and *parameters* could be very high-dimensional. This is also the drawback of RL where a lot of work is done to reduce the dimensions of state space and parameters. Interestingly, the instability is observed at the beginning of learning (Figure 5) then stability emerges from instability. Clearfield argues that new motor capabilities of infants emerge from instabilities (Clearfield, 2004, 2011; Clearfield et al., 2008). In Thelen’s theory, instability, including non-linearities, or phase shift or phase transition, is considered as the very source of new forms. In our implementation, the instabilities caused by exploration of an RL algorithm exactly leads to the final generation of a stable gradient. From the perspective of RL, instabilities in DST or infant learning may be the effects of preliminary exploration in order to seek the right direction of developmental tendency. Since the human body is an extremely sophisticated dynamic system which includes different levels (from microscopic to macroscopic) of high-dimensional parameter and state space, it takes more time and gets through more instabilities to finally converge to new behaviors. From the point of view of robotics, it also should be necessary to think about how a robot is able to learn in high-dimensional space with more intelligence. In this sense, cpg-actor-critic proffers a way to explore this open question of RL in a continuous space.

Interaction is of importance in locomotion learning. Inspired by infants learning to walk, the authors tested the use of assistive states (**X***_p_*) in cpg-actor-critic architecture. Since “Parental scaffolding” is a necessary factor helping infant to stand up and learn to walk through a repeated process (Adolph et al., 2012), the proposed architecture also shows possibilities of external assistance in learning to walk. Firstly, the assistive states which are directly related to the posture of ankles and knees could be interpreted as external force or bias. Hence, these states could be representations of outer assistance, e.g., from parents’ help. Secondly, infants start to learn to walk without mature value or emotion systems to evaluate their behaviors, parents play roles as infants’ emotion systems telling them which is good or not thereby causing the maturation of their affective systems (Schore, 2012). In RL, different rules of learning (like update rules and avoidance of falling) are adopted to place a “scaffolding” function primarily in a learning process. However, it lacks a general and evolvable value system for different types of locomotion learning. In this article, the value function is fixed and task-oriented working as a stability indicator for walking. In modern RL approaches, except dealing with more complex high-dimensional learning tasks, a generic reward system which can be adaptive to dissimilar situations is also a challenge. This is why a mature emotion system is demanded in a lot of robotic learning applications (Breazeal and Scassellati, 1999).

### 4.3 Conclusion and future work

In a nutshell, the work presented in this article simply shows the typical features of dynamic systems pertaining to instabilities, non-linearities, and adaptability to the environment. However, there is still a big difference in performance between an artificial, and a biological (human) adaptive dynamic system which solves more general problems in development and learning. Dynamic systems theory focuses on the development of systems in which new behaviors or attractors can emerge, disappear, and be memorized. In terms of this, RL, as a solver of general learning and developmental problems, needs further research.

In future work, we would like to test the results or the learning process on the physical NAO robot. Moreover, in order to testify the generality of our work and extend the adaptation of our model, experiments on different morphologies, and walking path planning (emphasized by Laumond; Arechavaleta, 2008; Mombaur et al., 2010) are also necessary.

## Conflict of Interest Statement

The authors declare that the research was conducted in the absence of any commercial or financial relationships that could be construed as a potential conflict of interest.

## Supplementary Material

The Supplementary Material for this article can be found online at http://www.frontiersin.org/Neurorobotics/10.3389/fnbot.2013.00005/abstract

## Footnotes

^1^The 2D or 3D means a coordination system fixed on the torso of a robot. It has three axes: X (Pitch: pointing to front), Y(Roll: pointing to right), Z(Vertical: pointing upwards).

## Appendix

### A The Details of CPG-Actor-Critic in the Implementation

#### A.1 Directions of flexor and extensor for each joint

In the pitch motion, there are two kinds of moving directions for each joint of NAO: forward (F) and backward (B). The directions of extensor and flexor are given: (1) Hip: Flexor (B+) and Extensor (F−). (2) Knee: Flexor (F+) and Extensor (B−). (3) Ankle: Flexor (B+) and Extensor (F−). The “−” and “+” represent the decrease and increase of joint values.

#### A.2 Details in RL and CPGs

In the RL, the policy parameters $\theta ∼\left[\theta_{\text{1:6}},\;\theta_{p12},\;\theta_{\text{9}}\right]$ are the weights **W** in CPGs (θ_9_ is not shown in the main text as it is not related to CPGs). The state space is $X∼\left\{X_{E}X_{F}X_{p}\right\},$ where $X_{E}∼\left\{X_{E1}X_{E2}X_{E3}\right\},\;X_{F}∼\left\{X_{F1}X_{F2}X_{F3}\right\},\;X_{p}=\left\{1,\;1\right\}.$ All the **X***_E_* and **X***_F_* are sensory feedback on sensor neurons with the functions given by: $\rho_{sn}=sigmoid\left(\theta_{threshold},\theta_{input},a\right)=\frac{1}{1+e^{a\left(\theta_{threshold}-\theta_{input}\right)}}.$ Then the Ū of RL policy could be written in details:

(A1) $$\begin{array}{lll} {Ū}_{E1} & =\theta_{1}X_{E1},\;{Ū}_{F1}=\theta_{2}X_{F1} & \end{array}$$

(A2) $$\begin{array}{lll} {Ū}_{E2} & =\theta_{3}X_{E2},\;{Ū}_{F2}=\theta_{4}X_{F2} & \end{array}$$

(A3) $$\begin{array}{lll} {Ū}_{E3} & =\theta_{5}X_{E3},\;{Ū}_{F3}=\theta_{6}X_{F3} & \end{array}$$

(A4) $$\begin{array}{lll} {Ū}_{p1} & =\theta_{p1}X_{p1},\;{Ū}_{p2}=\theta_{p2}X_{p2} & \end{array}$$

where for hip pitch motion **X***_F1_* = *sigmoid* (P*_sh_*, P*h*, 0.5) and **X***_E1_* = *sigmoid* (P*sh*, P, −0.5) are the proprioceptive (PP) sensor neurons, the Psh and Ph are the initial value of hip joint of standing posture and the value of the joint sensor. These two not only adjust the posture of hip but also can increase or limit the motion of the flexor or extensor. For the knee part, **X***_F2_* = *sigmoid* (P*_sk_*, P*_k_*, 16) and **X***_E2_* = *sigmoid* (P*_sk_*, P*_k_*, 16) are the same anterior extremity sensors. The P*_sk_* and P*_k_* are the basic posture of knee and the joint value of knee, respectively. 16 indicate a quick reflex when the knee joint reaches the extremity. As for the ankle part, **X***_F3_* = Ξ*sigmoid* (0, P*_g_*, 8) and XE3 = Ξ*sigmoid* (0, P*_g_*, −8) are ankle sensor neurons. Ξ is a function which is equal to 1 when the foot contacts the ground and 0 when there is no contact. P*_g_* is the angle of upright body based on the gyro sensor. These neurons are used to adjust the motion of ankle joint adaptively to the inclination angle of the body and work like a simple vestibular system. Therefore, the final output of CPGs is: (1) Hip: τ_1_ = τ_E1_ − τ_F1_. (2) Knee: τ_2_ = τ*_E2_* + τ*_F2_* + W*_p1_X**_p1_*, where W_p1_ is equal to converged θ_p1_. (3)Ankle: τ_1_ = τ_E3_ − τ_F3_ + W*_p_2X**_p_2*, where W*_p2_* is equal to converged θ*_p2_*. The control signals U=Ū+δ, where δ is a vector containing exploration values generated by RL policy. All the abovementioned equations are implemented on one leg and the same is used on the other leg because of the symmetry.

The roll motion adopts sensor-driven CPGs. For the hip roll: τ*_hl_* = *sigmoid*(*P_shl_*, *P_hl_*, 28) − *sigmoid*(*P_shr_*, *P_hr_*, 28) and τ*_hr_* = *sigmoid*(*P_shr_*, *P_hr_*, 28)-*sigmoid*(*P_shl_*, *P_hl_*, 28) are the output of roll CPGs to left and right hip roll joints, where *P_shl_*, *P_shr_* are the standing posture of left and right hip pitch joints and *P_hl_*, *Phr* are the values of joint sensors for left and right hip pitch joints. The same mechanism is for ankle roll: τ*_al_* = *sigmoid*(*P_sal_*, *P_al_*, 28) − *sigmoid*(*P_sar_*, *P_ar_*, 28) and τ*_ar_* = *sigmoid*(*P_sar_*, *P_ar_*, 28) − *sigmoid*(*P_sal_*, *P_al_*, 28) are the output of roll CPGs to left and right ankle roll joints, where *P_sal_*, *P_sar_* are the standing posture of left and right ankle pitch joints and *P_al_*, *P_ar_* are the values of joint sensors for left and right ankle pitch joints.

In order to better and stably approximate Q function in RL, we use another value-function related basis function ψ = 0.1*F* to increase the stability of RL, where F is the joint value of hip. Since the Equation 27 *J* = *V*^π^(**x***_H_+1*) − *V*^π^(**x**_0_), where *V*^π^(**x***_H_+1*) is the prediction of future value function dependent on state *x_H_*. So by using θ_9_ψ to approximate *V*^π^(**x***_H_+1*) can increase the stability of RL. *V*^π^(**x**_0_) is the value function of the initial state which is a constant approximated by baseline.
